# Turning Patients’ Open-Ended Narratives of Chronic Pain Into Quantitative Measures: Natural Language Processing Study

**DOI:** 10.2196/80269

**Published:** 2025-11-25

**Authors:** Raquel Norel, Jennifer Gewandter, Zhengwu Zhang, Anika Tahsin, Chadi G Abdallah, John Markman, Zhiyao Duan, Guillermo Cecchi, Paul Geha

**Affiliations:** 1 IBM (United States) Yorktown Heights, NY United States; 2 Department of Anesthesiology and Perioperative Medicine School of Medicine and Dentistry University of Rochester Rochester, NY United States; 3 Department of Statistics and Operations Research University of North Carolina Chapel Hill, NC United States; 4 Department of Psychiatry University of Rochester Rochester, NY United States; 5 Menninger Department of Psychiatry & Behavioral Sciences Baylor College of Medicine Houston, TX United States; 6 Eli Lilly (United States) Chestnut Hill, MA United States

**Keywords:** chronic pain, pain narratives, natural language processing, semantic distance, digital phenotyping

## Abstract

**Background:**

Subjective report of pain remains the gold standard for assessing symptoms in patients with chronic pain and their response to analgesics. This subjectivity underscores the importance of understanding patients’ personal narratives, as they offer an accurate representation of the illness experience.

**Objective:**

In this pilot study involving 20 patients with chronic low back pain (CLBP), we applied emerging tools from natural language processing (NLP) to derive quantitative measures that captured patients’ pain narratives.

**Methods:**

Patients’ narratives were collected during recorded semistructured interviews in which they spoke about their lives in general and their experiences with CLBP. Given that NLP is a novel approach in this field, our goal was to demonstrate its ability to extract measures that relate to commonly used tools, such as validated pain questionnaires and rating scales, including the numerical rating scale and visual analog scale.

**Results:**

First, we showed that patients’ utterances were significantly closer in semantic space to anchor sentences derived from validated pain questionnaires than to their antithetical counterparts. Furthermore, we found that the semantic distances between patients’ utterances and anchor sentences related to quality of life were strongly correlated with reported CLBP intensity on the numerical rating and visual analog scales. Consistently, we observed significant differences between individuals with low and high pain levels.

**Conclusions:**

Although our small sample size limits the generalizability of these findings, the results provide preliminary evidence that NLP can be used to quantify the subjective experience of chronic pain and may hold promise for clinical applications.

## Introduction

The most recent 2020 International Association for the Study of Pain definition of pain emphasizes that pain is “always a personal experience” and is distinct from nociception, involving learning “through life experiences.” In line with this definition, the biopsychosocial model of pain [1] also stresses that pain is “a personal experience emerging from the dynamic interplay between biological, psychological, and social factors.” Despite this modern understanding, the everyday clinical approach to pain still predominantly focuses on its location or injury-related causes, while monitoring pain primarily through subjective reports [2]. The “injury model” approach is incomplete, as it overlooks the affective and personal dimensions of the pain experience, which can have a significant negative impact on treatment response and long-term outcomes [3-5].

Chronic low back pain (CLBP), the most common chronic pain condition, is a typical example of the inadequacy of the “injury model” in explaining the origin of pain, the long-term outcomes, or response to treatment [5-7]. In fact, considerable variability exists among patients with low back pain, yet the most common subdivision still relies on peripheral symptom assessment, such as back pain with and back pain without radiculopathy [8,9]. The lack of understanding of this variability makes generalizing results from clinical trials to clinical practice very challenging because the observed effects in a heterogeneous population of patients do not necessarily translate to individual patients with specific phenotypes [10]. Clinical approaches that are able to capture different aspects of the “personal experience” of back pain, without relying solely on the traditional medical examination, are therefore needed to improve precision in characterizing and, consequently, treating subgroups of patients [11,12]. Experts agree that the absence of such approaches may be one of the reasons for the disappointingly poor outcomes of clinical trials for chronic pain conditions [13,14]. In addition, if these markers are affordable and easily implemented in a clinical setting, they could help generalize research results to clinical practice [13].

To date, there are no automated, quantitative tools that can both reliably assess patients with chronic pain [15] and provide clinicians with timely, clinically useful information about their pain experience. Rather, patients rely on verbal and nonverbal communication to convey their complaints. In everyday clinical practice, tools such as the numerical rating scale (NRS, 0-10), or self-administered pain questionnaires, including the McGill Pain Questionnaire (MPQ) [16] or the Patient-Reported Outcomes Measurement Information System (PROMIS) instruments, may be used. However, their utility is limited by the time constraints of busy clinical settings and by the restricted scope of information they provide. For example, an NRS rating of 4/10 does not capture the differences in the pain experience between a patient with low back pain and one with knee osteoarthritis. Therefore, patients rely on language to convey these differences to clinicians.

The language patients use can provide a valuable window into their personal experience of pain and the emotions associated with living with it. The recent advent of large language models (LLMs), which can analyze human-generated texts, offers a unique opportunity to probe the different dimensions of the chronic pain experience. Quantitative approaches of this kind are now beginning to emerge. LLMs have been applied to the analysis of clinical notes [17,18] and patients’ pain narratives [19-21]. For example, one study on patients with CLBP [20] investigated the language features that distinguished between responders and nonresponders to placebo treatment using a posttreatment interview. Another study analyzed written narratives obtained from patients with fibromyalgia and found significant agreement between LLM-based assessments and those of expert clinicians, supporting the use of LLM in the clinical evaluation of patients with pain.

Here, we used patients’ personal “pain stories” obtained during recorded interviews, along with natural language processing (NLP) tools, to derive quantitative measures of their pain experiences. We aimed to provide evidence that NLP can be used to analyze patients subjective accounts of their personal pain experiences and that the derived linguistic features are directly related to core phenotyping domains assessed using measures recommended by the Initiative on Methods, Measurement, and Pain Assessment in Clinical Trials (IMMPACT) and the National Institutes of Health minimal dataset for CLBP [13,22].

The interviews can be conducted in both clinical and nonclinical settings, and they are relatively inexpensive to obtain, thereby increasing accessibility for a broader patient population. The “pain story” is elicited through open-ended questions by a laboratory member without formal clinical training. It aims to capture not only a description of the physical symptoms but also how pain affects the patient’s functionality, interactions with others, and overall life trajectory and meaning. This rich dataset expands upon the information typically collected during clinical encounters, which are often constrained by time.

NLP is a novel approach for identifying or distinguishing patients with various psychiatric disorders, such as major depression or psychosis, where standard diagnosis relies on subjective reports of symptoms and the impression of clinical experts [23]. It involves a set of tools built using an extensive corpus of language available on the World Wide Web and machine learning methods to determine the various aspects (eg, meaning and emotion expressed) of words and sentences used in a text by situating them in the multidimensional linguistic space. NLP has been successfully used to distinguish patients with various mental health and neurological conditions [24-27].

## Methods

### Participants

This study was not registered before data collection. Data were collected between December 15, 2022, and June 8, 2023. A total of 514 individuals responded to advertisements posted on social media and flyers distributed within the Rochester community. Of these individuals, 21 patients with CLBP were recruited for this study between February and June of 2023; however, 1 patient did not show up for the testing session.

Patients were first screened over the phone. To be included, they had to be aged ≥40 years; this cutoff was chosen because the prevalence of low back pain is highest in individuals aged 40 to 80 years [28]. Patients had experienced low back pain for more than 1 year below the thoracic T10 vertebra [12], with an average pain intensity greater than 4/10 on the NRS. CLBP must be the primary pain condition. At screening, patients completed the Telephone Interview for Cognitive Status (TICS) [29] and were excluded if their TICS score was less than 33. We used TICS as a screening tool to avoid bringing patients to the laboratory who might have cognitive impairments that were not detected during the screening phone call. Patients were also excluded if they had a history of other significant pain conditions, such as fibromyalgia or migraine headaches; chronic unstable medical conditions affecting any bodily organ system; a history of traumatic brain injury; substance misuse; or mental illness. All patients, except 1, reported less than mild depression symptoms on the Montgomery-Asberg Depression Rating Scale (score *<*19) [30]. Patients were either opioid-naive or had not taken opioids for more than 2 weeks in the previous year. Table 1 presents the clinical and demographic characteristics of the CLBP sample; additional demographic and clinical characteristics are provided in Table S1 in Multimedia Appendix 1.

**Table 1:** Demographic and clinical characteristics (N=20, 10 males and 10 females).

Measure	Values, mean (SEM)
Age at screening (y)	58.3 (2.4)
BMI (kg/m^2^)	29.9 (2.1)
Years of education	14.9 (0.6)
NRS^a^ pain	4.0 (0.7)
VAS^b^ pain	35.1 (6.1)
MPQ^c^ total score	13.2 (2.8)
MPQ sensory score	11.1 (2.1)
MPQ affective score	2.2 (0.8)
PCS^d^	11.6 (4.1)
PDQ^e^	4.2 (2.1)
CSI^f^	33.9 (5.3)
VAS depression	16.1 (5.8)
MADRS-S^g^	7.9 (1.9)
HADS^h^-depression	4.4 (1.4)
HADS-anxiety	6.2 (1.2)
RMDQ^i^	8.9 (1.5)
TICS^j^	39.0 (1.0)
NEO^k^ personality inventory	200.1 (7.1)

^a^NRS: numerical rating scale.

^b^VAS: visual analog scale.

^c^MPQ: McGill Pain Questionnaire.

^d^PCS: Pain Catastrophizing Scale.

^e^PDQ: PainDETECT Questionnaire.

^f^CSI: central sensitization inventory.

^g^MADRS-S: Montgomery-Asberg Depression Rating Scale–Self-Report.

^h^HADS: Hospital Anxiety and Depression Scale.

^i^RMDQ: Roland-Morris Disability Questionnaire.

^j^TICS: telephone interview cognitive status.

^k^NEO: neuroticism, extraversion, and openness.

### Ethical Considerations

All participants provided written informed consent, and the study was approved by the University of Rochester Medical Center Institutional Review Board (00007633). Data protection was ensured using several layers of security in accordance with the Health Insurance Portability and Accountability Act and under the oversight of the University of Rochester Institutional Review Board . Participants were instructed not to mention their names during the interview, and their data were deidentified. All computer-based materials and data, including voice and video recordings, were stored in a password-protected server managed by the University of Rochester IT Department and protected by the university firewall. All clinical and demographic data were entered directly by the participants into the REDCap (Research Electronic Data Capture; Vanderbilt University), where the code numbers were associated with the data records. REDCap is a free, Health Insurance Portability and Accountability Act–compliant web-based application used for the electronic management of research data.

### Data Collection

#### Interview

On the day of the interview, after obtaining consent, participants provided a urine sample to screen for drugs of abuse using a NexScreen LLC drug screen cup. Drug screening was performed because drugs of abuse are known to alter pain perception [31,32] and language output [33,34], and misuse was one of our exclusion criteria. Next, the research coordinator reviewed the inclusion and exclusion criteria with the participants and explained the study procedures. Interviews were conducted via Zoom (Zoom Video Communications, Inc) to capture voice recordings on separate channels for the patient and the interviewer. Patients were seated in front of an iPad in a quiet room, while the coordinator was in an adjacent room. All patients were interviewed in the same room by the same female coordinator (AT), with a male laboratory member present in the coordinator’s room during the interviews. We included both male and female examiners during the interview to minimize bias in the patients’ responses related to sex. Although this bias has not yet been examined in the context of language-based studies, in other pain-testing contexts, the sex of the examiner has been shown to influence pain-related outcomes [35-37].

The interview questions, provided in Table S2 in Multimedia Appendix 1*,* served as a guide for the interviewer. The questions were piloted by AT, JG, and PG during the mock interviews. PG is a trained psychiatrist with extensive experience in interviewing patients in clinical settings. However, the interviewer did not have to adhere strictly to the interview questions and asked patients open-ended questions to elicit a subjective, patient-centered narrative of pain. As shown in Table S2 in Multimedia Appendix 1, the questions were inspired by previous NLP work in chronic pain [20] and by the general biopsychosocial model of chronic pain [1].

#### Questionnaires Collected

The patients completed the TICS and Montgomery-Asberg Depression Rating Scale over the phone as described earlier. All remaining pain ratings and questionnaires were completed after the interview to avoid any influence from these tools on the content of the interview. When in the laboratory, the patients rated their low back pain intensity on a visual analog scale (VAS) using a cursor on a line projected on the computer screen in front of them and completed the National Institutes of Health minimal dataset for CLBP [22]. They also completed questionnaires and measures recommended by IMMPACT [13], including the short form of the MPQ, the PainDETECT Questionnaire, the Pain Catastrophizing Scale, the Hospital Anxiety and Depression Scale, and the Roland-Morris Disability Questionnaire. The core domains recommended by IMMPACT overlap extensively with those identified in a more recent consensus report on core outcome sets [14]. In addition, the patients with CLBP completed the central sensitization inventory, as well as the neuroticism, extraversion, and openness personality inventory, and reported their list of prescribed medications.

### Preprocessing of Interview Text

Voice data were recorded in 2 channels, one for the interviewer and one for the participant. Automatic audio transcription of the entire participants’ interview was performed using the Whisper base model [38]. We used the transcriptions verbatim for further analysis and performed the analysis sentence by sentence. Sentences were obtained using the sentence tokenizer from the Natural Language Toolkit [39], which is an open-source Python library designed for working with human language data.

### Data Analysis

#### Construction of Anchor Sentences

Our aim was to understand how the patients’ personal accounts of their “pain stories” were situated in a multidimensional linguistic space relative to the core domains and validated measures recommended by experts’ consensus [13,22]. These measures are often used in chronic pain research but are less commonly applied to monitor patients in everyday clinical settings. To achieve this, we constructed anchor sentences based on validated questionnaires that covered topics related to the sensory, affective, functional, and quality-of-life aspects of patients with CLBP. We present all the anchor sentences that we used in Table S3 in Multimedia Appendix 1*,* along with the source questionnaire from which they were derived. The questionnaires included the MPQ [16], the Pain Catastrophizing Scale [40,41], PROMIS [42,43], and the World Health Organization Quality of Life Questionnaire [44], which are measures tested for content validity and involved patients’ population during their conception. Because LLMs can misunderstand negation [45,46], some of the anchor sentences were rephrased from the original questionnaires. Although the list of anchor sentences may not be exhaustive, we ensured that they addressed the biological, psychological, and social domains of patients’ lives.

#### Feature Extraction

**Figure 1:**
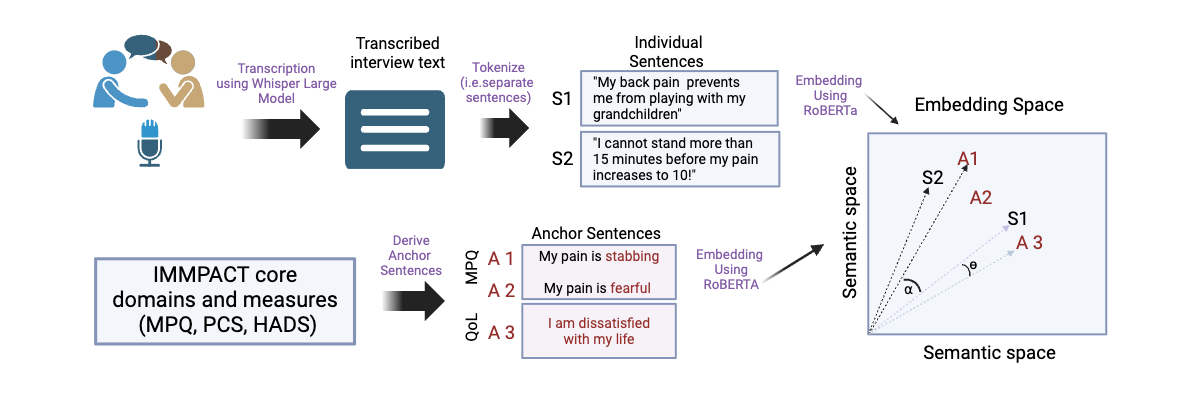
Schematic representation of the data collection and analysis workflow. Transcribed interview text and anchor sentences were semantically embedded to calculate the semantic similarity between the anchor sentences and the interview text. The embedding space represented here is a projection to a 2D graph and is therefore a simplification of the actual space, which comprises 1024 dimensions. As depicted, sentence S1, uttered by the patient about daily life, is closer to anchor A3 than to anchor A1 (ie, θ<α). HADS: Hospital Anxiety and Depression Scale; IMMPACT: Initiative on Methods, Measurement, and Pain Assessment in Clinical Trials; MPQ: McGill Pain Questionnaire; PCS: Pain Catastrophizing Scale; QoL: quality of life.

We used semantic embedding [47], a fundamental NLP and machine learning concept, for semantic feature extraction from the interview text. Figure 1 illustrates the overall approach. This method consists of projecting entire sentences into a semantic space and converting them to vectors with 1024 coordinates [48]. It enables computers to understand and process human language by mapping words, phrases, or entire documents to vectors of real numbers. These vectors can then be analyzed with a rich set of mathematical tools to extract valuable information from the text, facilitating tasks such as sentiment analysis, topic modeling, and semantic similarity measurement, which we used in this work [49-52]. We generated semantic embeddings using the RoBERTa (a robustly optimized bidirectional encoder representations from transformers pretraining approach) large model [48], which was trained on 160 GB of text.

#### Analysis Using Anchor Sentences and Their Antithetical Pair

We wanted to evaluate whether the anchor set, derived as described in the previous section, adequately covered the interview topics by comparing the semantic distance between the interview sentences and both the anchor and its antithesis. More specifically, we calculated the semantic embeddings (Figure 1) of each sentence uttered by the patient—using the entire interview—the anchor sentence, and its antithetical pair. Next, semantic similarity was quantified by calculating the cosine similarity between the vectors of the anchor sentences and interview sentences, on the one hand, and the antithetical anchor sentences and the interview sentences on the other hand, providing a quantitative measure of their semantic resemblance. This calculation resulted in 2 distributions of semantic similarities for each anchor pair. We used the 95th percentile point (Figure S2 in Multimedia Appendix 1) of each distribution to represent the semantic distance of the interview sentences to the anchor and to its antithetical pair, respectively. This value, the 95th percentile of the similarity scores, represents a robust maximum after omitting outliers. The robust maximum is more resistant to outliers than the absolute maximum and is inherently invariant to the length of the interview. To further illustrate our approach, we plotted the distribution of semantic similarities from 2 participants representing the extreme ends of the number of sentences uttered during the interview to demonstrate the robustness of the 95th percentile, which falls within the 0.4 to 0.6 range of semantic similarity (Figure S1 in Multimedia Appendix 1).

#### Relating Semantic Similarities to Reported Back Pain Intensity

Ratings of chronic pain intensity are often used in clinical settings and are a mainstay of clinical pain research. Therefore, we tested the relationship of semantic similarities to the anchor sentences, showing significant differences in the previous analysis and the pain intensity reported on the NRS and the VAS. We calculated the correlation between semantic similarities to the anchor sentences, showing significant differences between the previous analysis and the reported pain back pain ratings. We also assessed whether the anchor sentences were sensitive enough to detect differences between interviews from the high- and low-pain subgroups.

#### Statistical Analysis

The Kolmogorov-Smirnov (KS) test was used to assess the differences between the distributions of semantic similarities. Specifically, we compared the distribution of the 95th percentile robust maximum for each anchor sentence with that of its corresponding antithetical sentence across patients. Because we analyzed 33 pairs of sentences, we corrected for multiple comparisons using the false discovery rate (FDR) method. Additionally, we compared the distributions of semantic similarities to the anchor sentences between the high-pain and low-pain groups using the KS test. Finally, we used Spearman’s ρ correlation coefficient to examine the relationship between semantic similarities and reported pain intensity ratings.

## Results

### Demographic and Clinical Characteristics

Table 1 shows the demographic and clinical characteristics of the sample of 20 patients with CLBP. In total, 50% of the participants were female. By design, the patients did not have any evidence of cognitive impairment (*TICS>*33). Complete details of the patients’ characteristics are reported in Table S1 in Multimedia Appendix 1.

### Analysis of the Interviews

#### Interview Duration

**Figure 2:**
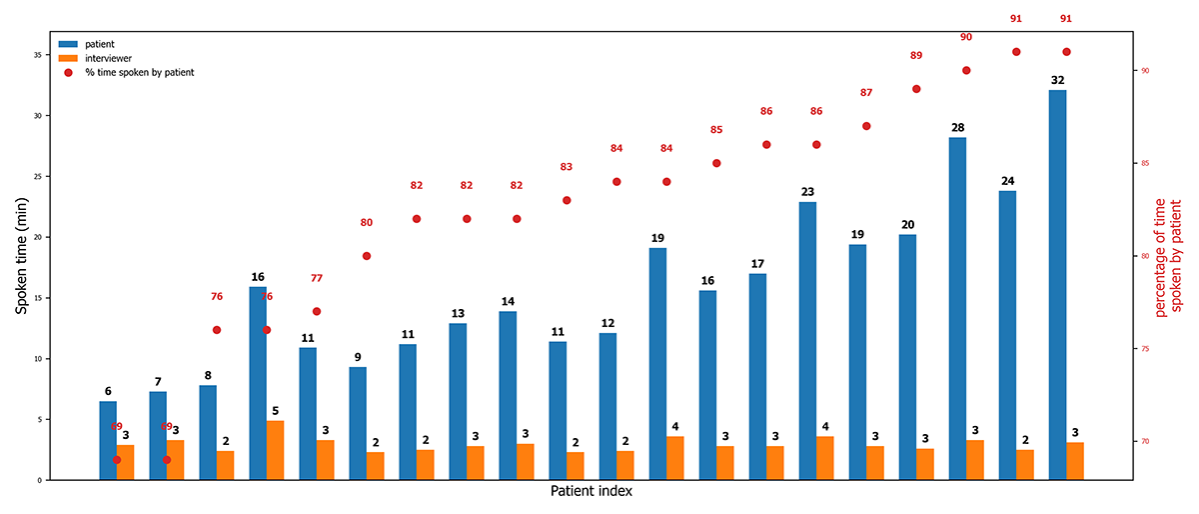
Interview duration and percentage of time spoken by the interviewer and the patient. The left axis depicts the duration (in minutes) spoken by each patient (blue) and the interviewer (dark orange); the right axis depicts the percentage of interview time spoken by the patient (red dots). The x-axis depicts all patients (N=20), arranged by interview duration from shortest to longest.

The duration of the interviews ranged from less than 10 minutes to more than half an hour, with a total of 376.4 minutes of speech. The interviews across all participants included a total of 43,641 words of naturalistic language data with a unique vocabulary of 3158, making the total sample a substantial linguistic corpus. The vocabulary richness ratio was 0.072, and the natural speech rate was 115.9 words per minute, demonstrating genuine and varied linguistic expression rather than constrained responses [53,54]. The patients spoke between 69% and 91% of the interview time (Figure 2). There was no statistically significant correlation between pain scores and interview duration (NRS and VAS: *P*=.94) or between pain scores and the percentage of time spoken by the participant (NRS: *P*=.42; VAS: *P*=.72). Because years of education may affect speech generation or the richness of the text generated, we examined the correlation between years of education and the interview features, such as duration, time spoken by the participants, and time spoken by the interviewers, as well as between years of education and the semantic embeddings of the anchor sentences (Table S4 in Multimedia Appendix 1). Years of education were negatively correlated with the time spoken by the interviewer, indicating that the latter prompted participants with fewer years of education more frequently than those with more years of education. Years of education were not correlated with the overall interview duration, the time spoken by the participant, the word content, or the pain scores. In addition, years of education were not significantly related to the semantic embeddings of the anchor sentences, except for “I rate my quality of life as poor” and “My physical environment is unhealthy.”

#### Semantic Distance of Interview Texts to Anchor Sentences and Their Antithetical Sentences

Figure S2 in Multimedia Appendix 1 illustrates the distributions of semantic similarities for 1 anchor sentence—“I have no pain”—and its antithesis—“I am in pain.” Table S5 in Multimedia Appendix 1 shows example semantic embeddings for 2 anchor sentences. The semantic embeddings of 7 pairs of anchor sentences and their antithesis showed statistically significant differences (*P*<.05; FDR corrected) in their similarities to interview sentences (Table 2). In total, 5 pairs of sentences were derived from the MPQ, 1 from the measure of pain variability, and 1 from the PROMIS-Pain Interference questionnaire. Table S6 in Multimedia Appendix 1 presents the results for all the anchor-antithesis sentence pairs tested.

**Table 2:** Anchors and their antithetical sentences showing significant differences in their semantic similarities to the interview text.

Anchor	Antithesis	Questionnaire	KS^a^ test	*P* value	q value^b^
My pain is cramping	My pain is relaxing	MPQ^c^	0.95	5.80×10⁻¹⁰	1.92×10⁻⁸
My pain is splitting	My pain is light and dull	MPQ	0.8	1.33×10⁻⁶	1.46×10⁻⁵
My pain is throbbing	My pain is steady	MPQ	0.65	2.70×10⁻⁴	2.23×10⁻³
My pain is stabbing	My pain is dull and aching	MPQ	0.65	2.70×10⁻⁴	2.23×10⁻³
My pain is tiring-exhausting	My pain is bearable	MPQ	0.55	3.97×10⁻³	2.18×10⁻²
I am in pain	I have no pain	Pain variability	0.85	1.43×10⁻⁷	2.37×10⁻⁶
I am unable to focus	I am able to focus	PROMIS^d^-pain interference	0.55	3.97×10⁻³	2.18×10⁻²

^a^KS: Kolmogorov-Smirnov.

^b^q value refers to the false discovery rate–adjusted *P* value. Each anchor sentence has its opposite (eg, I have no pain and I am in pain). We computed the semantic similarity between each sentence from the interview and the anchor. From that distribution of values (1 value per sentence), we retained the robust maximum (the 95^th^ percentile, which is more resistant to outliers than the maximum value). We then repeated the same calculation for the antithesis of the anchor, also retaining the robust maximum semantic similarity. We repeated this for each participant, resulting in 1 distribution of values extracted from the anchors and 1 from their antitheses. We then compared these two distributions using the 2-sample Kolmogorov-Smirnov test to determine whether significant differences existed between the anchor and its antithesis used to query the data in this cohort. This nonparametric test is designed to determine if 2 samples originate from the same distribution.

^c^MPQ: McGill Pain Questionnaire.

^d^PROMIS: Patient-Reported Outcomes Measurement Information System.

#### Relationship Between Semantic Distance of Interview Texts to Anchor Sentences and Back Pain Intensity

**Figure 3:**
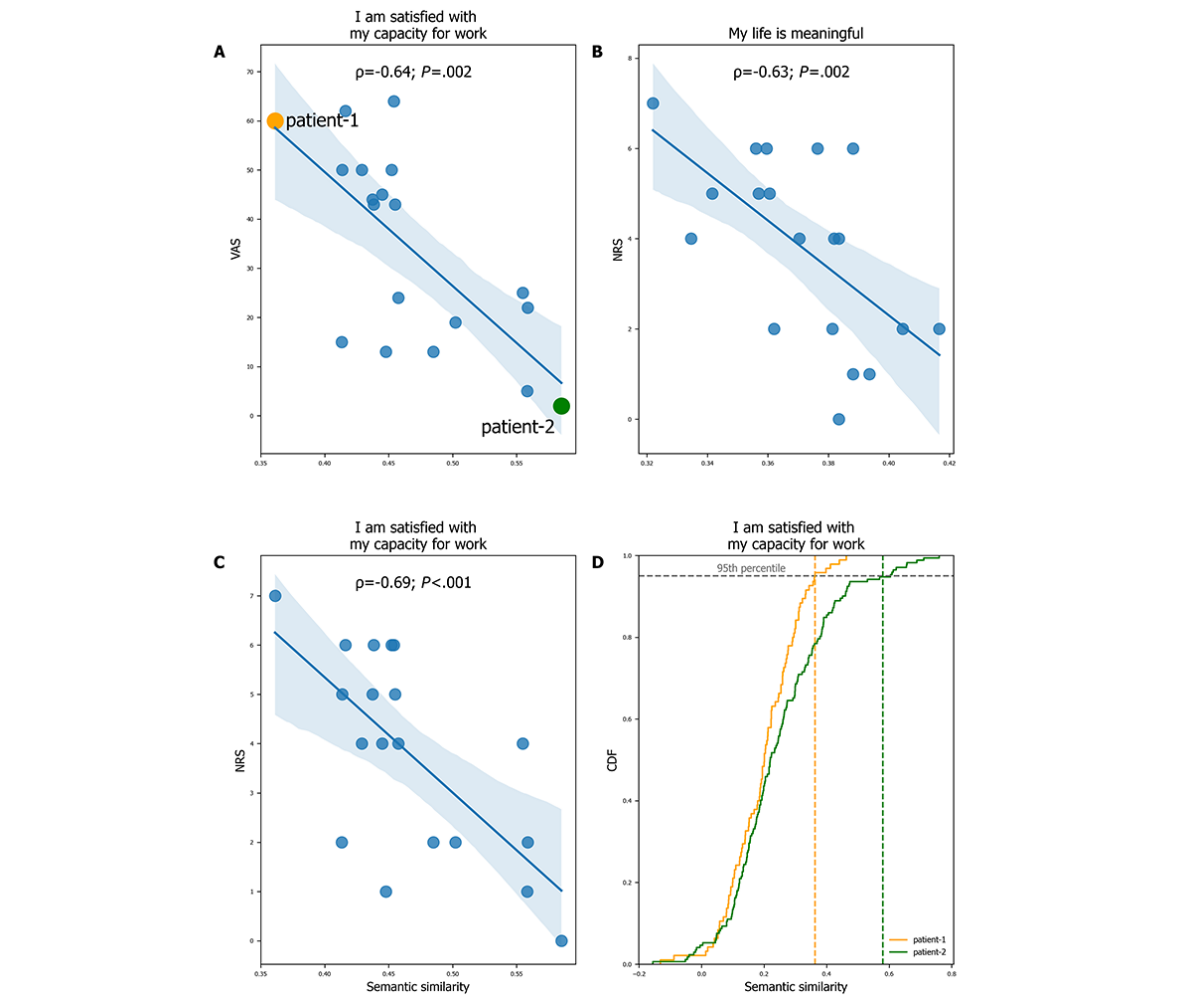
Relationship (Spearman ρ, *P*<.01) between the semantic similarity of patients’ texts and anchor sentences and the rated back pain intensity. Significant correlations between back pain intensity and semantic similarity for “I am satisfied with my capacity for work” (A and C) and “I am satisfied with my ability to perform my daily living activities” (B). (D) Examples of 2 cumulative distributions of semantic similarity between the interview text and “I am satisfied with my capacity for work” obtained from 2 patients on both sides of the pain intensity spectrum, as shown in panel A. Patient 1 (dark orange) experienced relatively higher low back pain intensity and therefore showed smaller similarity to the anchor sentence, whereas patient 2 (green) showed the opposite pattern. Dashed lines represent the 95th percentile (the robust maximum used in our statistical analyses). The blue shading represents the CIs for the regression line. CDF: cumulative distribution function; NRS: numerical rating scale; VAS: visual analog scale.

**Figure 4:**
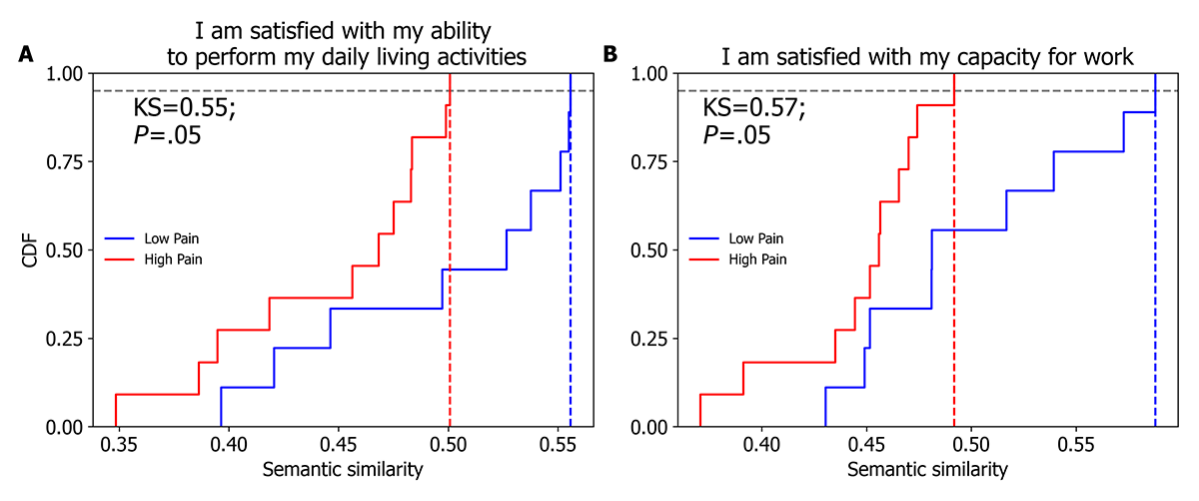
Comparison of the distribution of semantic similarities to anchor sentences between high (visual analog scale [VAS]>30; red) and low pain (VAS≤30, blue) groups. The cumulative distributions of semantic similarity between the interview text and “I am satisfied with my ability to perform my daily living activities” (A), and “I am satisfied with my capacity for work” (B), differed significantly and were shifted to the left in the high-pain group compared with the low-pain group (Kolmogorov-Smirnov [KS] test, *P*<.01). CDF: cumulative distribution function.

NRS and VAS are commonly used tools in clinical and research settings. Therefore, we tested whether the semantic distance of the patients’ interviews to the anchor sentences scaled with the patients’ reported pain intensity. Figure 3 shows 3 examples in which the correlations were statistically significant (*P*<.05; FDR corrected). The semantic similarity to the anchor sentence “I am satisfied with my capacity for work” was inversely related to both NRS and VAS scores. Similarly, the semantic similarity to “I am satisfied with my ability to perform my daily living activities” was inversely related to the NRS pain score. In the same figure, we illustrate the differences in the distributions of semantic similarity between the interview and the antithesis “I am satisfied with my capacity for work” for 2 participants: 1 with high pain (patient-1) and 1 with low pain (patient-2). Table S7 in Multimedia Appendix 1 presents all calculations performed between the anchor NRS and VAS, on the one hand, and the semantic embeddings of the anchor sentences, on the other hand. Notably, the NRS and VAS were most strongly correlated with the semantic embeddings of anchor sentences describing the quality of life. Another way of examining the relationship between the interview text and pain intensity was to compare the high- and low-pain subgroups. Patients who reported a VAS pain score *≤*30 or an NRS score *≤*3 were classified into the low-pain group; otherwise, they were classified into the high-pain group. Figure 4 shows the differences between the subgroups with high and low pain in the semantic similarity to “I am satisfied with my capacity for work” and to “I am satisfied with my ability to perform my daily activities.” Not surprisingly, participants with high pain reported less satisfaction with their quality of life (Figure 4).

## Discussion

### Principal Findings

In this study, we present evidence that subjective narratives of patients with CLBP, elicited through open-ended questions about their lives posed by an interviewer with no formal clinical training, can provide semantic features directly related to well-validated tools currently used to assess patients with pain [13]. Semantic embeddings, which assess the semantic similarity between anchor sentences—derived from validated pain and quality of life questionnaires—and sentences from the interviews, demonstrated statistically significant differences between negative and positive assertions about pain. In other words, the interview texts from patients showed asymmetric semantic similarities when queried with pain-related anchor sentences, providing evidence that NLP can detect clinically meaningful signals from the content of the interviews.

Additionally, the semantic embeddings differed significantly when their distributions were compared between subgroups with low and high pain levels. Notably, our analysis revealed a strong connection between the language used by patients with CLBP and their self-reported satisfaction with work capacity and other quality of life measures. Specifically, the semantic similarities of interview texts to anchors probing patient satisfaction with their life, such as “I am satisfied with my capacity for work” or “I am satisfied with my ability to perform my daily activities” were strongly inversely correlated with pain intensity, indicating that these aspects of patients’ lives are deeply intertwined with their experience of pain. The statement “I am satisfied with my capacity for work,” derived from the World Health Organization Quality of Life Questionnaire [44], reflects patients’ perception of how pain impacts their ability to perform work-related tasks, maintain employment, and engage in productive activities. Satisfaction with work capacity may indicate a degree of pain management and coping strategies that enable individuals to continue participating in meaningful and fulfilling roles despite their condition [55]. Both anchors underscore the importance of addressing not only the physical symptoms of pain but also its broader psychological and existential impacts. This also suggests that patients’ perception of their ability to work can serve as an indicator of their overall well-being and a potential target in chronic treatment. This finding aligns with the results of the INTEGRATE-pain Delphi process [14], which identified activities of daily living and quality of life as core outcome measures for chronic pain treatment. Furthermore, it highlights how applying NLP to patient interviews can extract patient-centered, quantitative measures that are not readily available from current state-of-the-art assessments in busy clinical settings, where the focus tends to be more on the sensory aspects of pain.

Not all anchor-antithesis pairs derived from validated questionnaires showed a statistically significant difference in their semantic distance to patients’ narratives. In total, 21 of the 33 anchor pairs yielded a KS value ≥0.3, indicating a moderate-to-large separation between the distributions of semantic distances [56]. We predict that all these pairs will reach statistical significance with larger samples. The remaining 12 pairs had a KS value <0.30, indicating only a small separation. Several factors may account for these findings. These pairs may not adequately capture the nuances of the interview texts, or the way we constructed the pairs may have been limited. Another possibility is that the RoBERTa large model [48] used for semantic embeddings has intrinsic limitations. As these models continue to evolve, their embeddings are refined, potentially enhancing their ability to capture the relationships between questionnaire-based anchors and patients’ narratives.

To date, there are no validated and widely accepted automated tools that can reliably assess patients with chronic pain; therefore, these patients must rely on verbal and nonverbal communication to convey their distress to others, including clinicians. Having such tools is critical for guiding clinical decision-making and for conducting valid, quantitative research on the mechanisms and therapeutics of chronic pain. Until recently, research on pain language has remained qualitative [57] or was mostly limited to single-word descriptors embedded in questionnaires such as the well-known MPQ [16]. Nevertheless, studies using quantitative linguistic analysis of narratives from patients with chronic pain to measure pain or response to interventions have begun to emerge rapidly [20,21,58]. We have previously shown, for example, that by using LLMs to extract linguistic features from patients’ interviews, we could distinguish between those with fibromyalgia and those with neuropathic pain, with an area under the receiver operating characteristic curve of 0.83 despite limited sample sizes [59]. Furthermore, we have demonstrated that NLP applied to patients’ narratives can retrospectively [21] and prospectively predict response to treatment [20,58]. Nunes et al [60] analyzed short interviews recorded in response to 7 questions about patients’ pain from 65 participants with various chronic pain conditions who reported mild, moderate, or severe pain. An NLP-based support vector machine model with early fusion of linguistic features was able to distinguish among the 3 groups of patients, thereby demonstrating, in a proof of concept, that NLP can be used to estimate chronic pain intensity. This emerging field, combined with the ease of obtaining voice recordings through the widespread use of smart devices, has the potential to provide novel, automated, and quantitative measures of patients’ experiences of pain. These measures can be assessed frequently and can complement existing tools to enhance both patient care and research.

A review of the recent literature [61] studying pain language analysis using computational methods found that most studies relied on electronic health records and social media posts, with only a minority relying on patients’ interviews. The most common objective was to classify patients with chronic pain. Our aim in this study was to demonstrate that a relatively brief interview (*<*30 min), inspired by the biopsychosocial assessment of pain and designed to elicit a subjective narrative of the patient’s daily life and pain experience, can be analyzed using NLP tools to capture quantitative and clinically significant measures of the pain experience. These measures can then be used in future studies using the same approach to differentiate among patient groups, monitor progress, or evaluate treatment response. In addition to being patient-centered, substantial advantages of this approach include the ability to obtain data remotely and the absence of the need for a medical professional during data collection, both of which significantly reduce the cost of data collection.

### Study Limitations

This study included a small sample of patients aged ≥40 years, which may limit the generalizability and linguistic representativeness of the findings. In addition, the small sample may have included subgroups of patients with CLBP—such as those with or without a neuropathic component—which could introduce heterogeneity into both the pain experience and the language used by the patients. Furthermore, the semantic distance calculations do not fully capture the complete meaning, with all its nuances, expressed by the patients. Thus, NLP cannot replace expert judgment but should rather be used alongside expert clinician assessments. Additionally, the process of constructing anchor sentences—particularly given the inability of LLMs to handle negations—may have affected the content validity of the anchors used, despite their derivation from content-valid questionnaires. Nevertheless, the language features related to validated pain questionnaires were strongly correlated with reported pain intensity and significantly distinguished between the high- and low-pain groups. Future research should explore larger and more diverse cohorts of patients with CLBP, including more representative age groups and groups with varying levels of common CLBP comorbidities such as depression, anxiety, or cognitive changes, to confirm these findings and investigate other aspects of the condition, such as its progression or response to treatment.

## Data Availability

The datasets generated or analyzed during this study are not publicly available due to participants’ confidentiality.

## Appendix

Multimedia Appendix 1 Additional methods and tabulated data.

## Abbreviations

CLBP: chronic low back pain
FDR: false discovery rate
IMMPACT: Initiative on Methods, Measurement, and Pain Assessment in Clinical Trials
KS: Kolmogorov-Smirnov
LLM: large language model
MPQ: McGill Pain Questionnaire
NLP: natural language processing
NRS: numerical rating scale
PROMIS: Patient-Reported Outcomes Measurement Information System
REDCap: Research Electronic Data Capture
VAS: visual analog scale
