# Supplementary material for: Turning Patients’ Open-Ended Narratives of Chronic Pain Into Quantitative Measures: Natural Language Processing Study
Source: JMIR Hum Factors. 2025 Nov 25;12:e80269. doi: 10.2196/80269 (PMC12690277; doi:10.2196/80269)
Supplement: Multimedia Appendix 1 [file humanfactors_v12i1e80269_app1.docx]

**Multimedia appendix 1**

**Supplementary Methods**

1. **Distribution Comparison and Anchor Sentences**

To obtain the distribution of semantic similarities between interview texts on the one hand, and anchor sentences and their antithetical version, on the other hand, we computed the cosine similarity of each of the interview text and the anchor sentence and its antithetical version. Because we want to determine if the individual referred to the topic in the anchor sentence, we retained the robust maximum (95th percentile of the distribution of values (**Figure S1**)). In other words, we retain the “closest” value in the semantic distance. We repeat the same process for the antithetical sentence. This results in two distributions of values; one corresponds to the robust maximums of the positive anchors, and one corresponds to the robust maximum of their antithesis across all patients. An example of such distribution is depicted in **Figure S2.**

**Supplementary Figures**

**
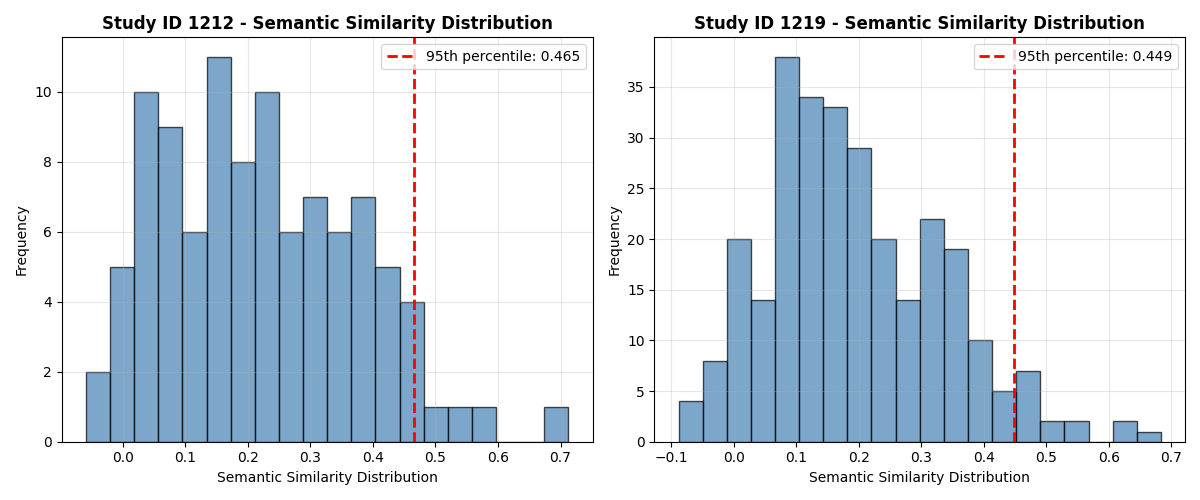
**

**Figure S1. Distribution of semantic similarities in two examples.** The left and right panels illustrate the distribution of semantic similarities of the interview sentences uttered by subject 1212 (left), who produced the smallest number of sentences (75 sentences), and subject 1219 (right), who produced the largest number of sentences (257), in our sample. The red dotted lines indicate the 95^th^ percentile. Despite the ~ 3 fold difference in the number of sentences between these 2 subjects 95^th^ percentile lies within a narrow range of semantic similarity (0.4-0.6).


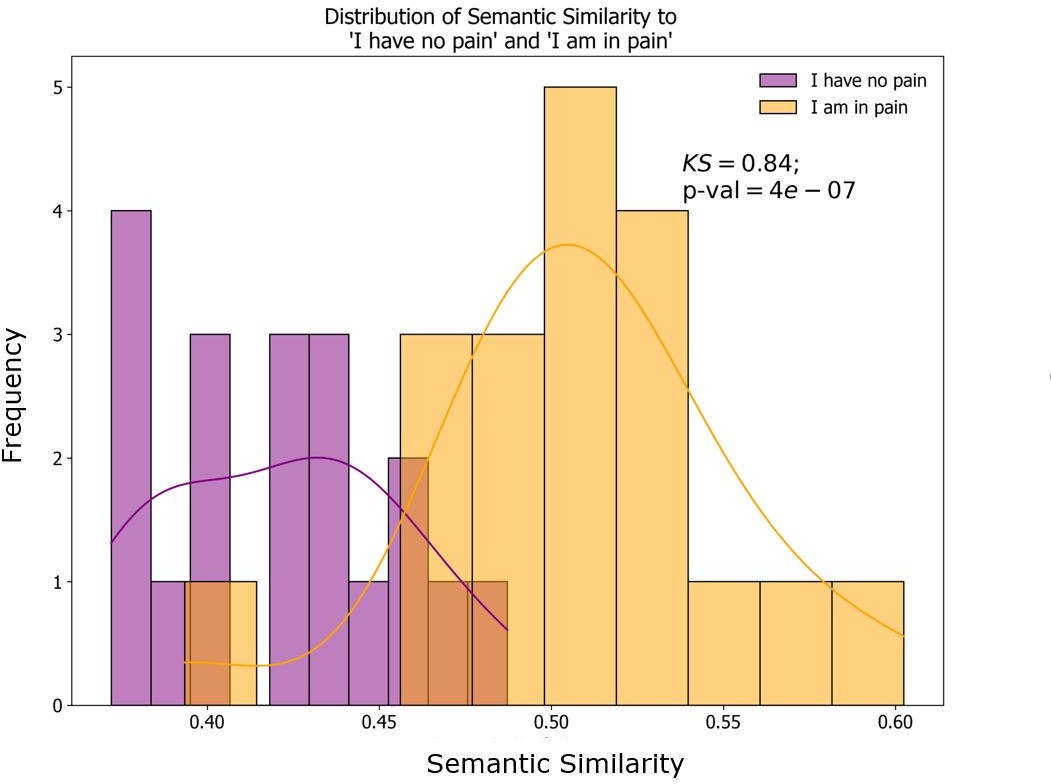
**Figure S2: Distribution of semantic similarity for a positive and negative version of an anchor phrase.** Sentences from patients have higher similarity to “I am in pain” than to “I have no pain”, as expected. Size effect, measured as Cohen’s d is 2.2, indicating a huge effect size. These examples are taken from real interviews.

**Supplementary Tables**

**Table S1:** Demographics and clinical data for all participants.

| **ID** | **Sex** | **Age** | **BMI**  **Kg/m^2^** | **Edu.**  **years** | **Dur.**  **years** | **NRS** | **VAS** | **TICS** | **MADRS** | **VAS Dep.** | **HADS**  **Dep.** | **HADSAnx.** | **PCS** | **PDQ** | **MPQt** | **MPQs** | **MPQa** | **RMDQ** | **NEO** | **CSI** |  |
| --- | --- | --- | --- | --- | --- | --- | --- | --- | --- | --- | --- | --- | --- | --- | --- | --- | --- | --- | --- | --- | --- |
| **1202** | F | 61 | 20.7 | 16 | 30 | 4 | 24 | 38 | 12 | 0 | 1 | 8 | 11 | 0 | 27 | 20 | 7 | 7 | 224 | 28 |  |
| **1203** | F | 61 | 26 | 18 | 40 | 5 | 50 | 40 | 13 | 37 | 4 | 7 | 9 | 8 | 12 | 11 | 1 | 8 | 225 | 37 |  |
| **1204** | M | 57 | 26.7 | 16 | 30 | 1 | 13 | 39 | 18 | 16 | 7 | 8 | 16 | 0 | 14 | 14 | 0 | 6 | 190 | 57 |  |
| **1205** | F | 70 | 19.1 | 16 | 15 | 6 | 43 | 41 | 3 | 2 | 1 | 2 | 3 | 0 | 5 | 5 | 0 | 6 | 229 | 32 |  |
| **1206** | F | 60 | 18.5 | 16 | 29 | 4 | 45 | 38 | 6 | 46 | 3 | 11 | 31 | 6 | 15 | 9 | 6 | 7 | 206 | 50 |  |
| **1207** | F | 60 | 34 | 16 | 40 | 7 | 60 | 38 | 8 | 4 | 7 | 8 | 11 | 5 | 11 | 9 | 2 | 12 | 196 | 38 |  |
| **1208** | F | 44 | 22.1 | 18 | 2 | 5 | 43 | 38 | 15 | 14 | 6 | 13 | 1 | 0 | 3 | 3 | 0 | 4 | 203 | 46 |  |
| **1210** | F | 44 | 35.8 | 14 | 2.5 | 6 | 64 | 37 | 10 | 31 | 3 | 5 | 10 | 0 | 15 | 11 | 4 | 12 | 233 | 39 |  |
| **1211** | F | 48 | 43.4 | 14 | 20 | 4 | 50 | 42 | 17 | 50 | 11 | 4 | 4 | 10 | 23 | 18 | 5 | 12 | 165 | 67 |  |
| **1212** | M | 58 | 36.7 | 12 | 15 | 5 | 44 | 36 | 12 | 22 | 4 | 4 | 5 | 4 | 6 | 6 | 0 | 5 | 173 | 31 |  |
| **1213** | M | 52 | 29.2 | 16 | 1.5 | 2 | 19 | 41 | 2 | 10 | 1 | 5 | 14 | 4 | 17 | 16 | 1 | 5 | 208 | 27 |  |
| **1214** | M | 58 | 31.9 | 12 | 4 | 7 | 52 | 36 | 3 | 50 | 18 | 12 | 52 | 29 | 38 | 30 | 8 | 20 | 197 | 42 |  |
| **1215** | M | 58 | 33.9 | 16 | 15 | 0 | 2 | 37 | 0 | 0 | 0 | 0 | 0 | 0 | 12 | 12 | 0 | 16 | 208 | 12 |  |
| **1216** | M | 67 | 30.2 | 14 | 15 | 1 | 5 | 39 | 0 | 0 | 1 | 2 | 0 | 1 | 3 | 3 | 0 | 6 | 191 | 9 |  |
| **1217** | M | 64 | 26.3 | 16 | 16 | 2 | 13 | 45 | 2 | 3 | 1 | 4 | 8 | 0 | 8 | 6 | 2 | 5 | 210 | 12 |  |
| **1218** | M | 47 | 43.1 | 12 | 5 | 6 | 62 | 35 | 10 | 32 | 4 | 5 | 14 | 4 | 13 | 12 | 1 | 15 | 139 | 24 |  |
| **1219** | M | 61 | 52.0 | 14 | 42 | 2 | 15 | 35 | 16 | 2 | 7 | 4 | 28 | 10 | 14 | 11 | 3 | 12 | 181 | 48 |  |
| **13002** | M | 65 | 25.4 | 14 | 5 | 2 | 22 | 41 | 2 | 0 | 0 | 2 | 0 | 0 | 2 | 2 | 0 | 2 | 200 | 7 |  |
| **13003** | F | 65 | 21.0 | 14 | 2.5 | 4 | 25 | 37 | 6 | 0 | 4 | 13 | 13 | 0 | 13 | 12 | 1 | 9 | 223 | 40 |  |
| **13004** | F | 65 | 22.1 | 14 | 10 | 6 | 50 | 46 | 2 | 3 | - | - | 2 | 3 | - | - | - | - | - | 13 |  |
| *Abbreviations:* BMI, body mass index*;* Dur, duration; Dep, depression; Edu, education; CSI, central sensitization index; HADS, hospital anxiety and depression scale; MADRS, Montgomery-Asberg Depression Rating Scale (MADRS);MPQ, short form of the McGill Pain Questionnaire (t) total, (s) sensory, and (a) affective; NEO, neuroticism, extraversion, openness personality inventory; NRS, numerical rating scale; PCS, pain catastrophizing scale; PDQ, PainDETECT; RMDQ, Roland-Morris disability questionnaire; TICS, telephone interview cognitive status; VAS, visual analog scale. | | | | | | | | | | | | | | | | | | | | | |

**Table S2:** Questions asked in the interview.

| **Non-Pain Questions** | Tell me a little bit about yourself. |
| --- | --- |
|  | Tell me about things you enjoy doing. |
|  | Tell me about a recent dream that you can describe vividly. |
|  | Please describe a recent event you had with family and/or friends. |
| **Pain Related Questions** | What is your pain story? |
|  | What do you think is causing your back-pain? |
|  | What are things that affect your pain? |
|  | How does your pain affect your life? |
|  | How does pain affect your concentration? |
|  | How does pain affect your mood? |
|  | What treatment(s) have you tried for your pain? |
|  | How do you think you are doing compared to other people with  chronic low-back pain? |
|  | Do you see yourself differently from other people who have pain? |
|  | How has pain affected your relationship with other people? |
|  | Have you tried any alternative therapies? (Acupuncture? Neuro-  feedback? Aqua therapy? Yoga? Transcutaneous electrical nerve stimulation (TENS)? Massage? Chiropractice?) |
|  | Is there anything else you want to tell me so I can better understand  your pain experiences? |

**Table S3:** Anchor sentences and source

| **Anchor** | **Antithesis** | **Source** |
| --- | --- | --- |
| My pain is throbbing | My pain is steady | MPQ |
| My pain is shooting | My pain is dull and localized | MPQ |
| My pain is stabbing | My pain is dull and aching | MPQ |
| My pain is sharp | My pain is dull | MPQ |
| My pain is cramping | My pain is relaxing | MPQ |
| My pain is gnawing | My pain is mild and intermittent | MPQ |
| My pain is hot burning | My pain is numbing | MPQ |
| My pain is aching | My pain is fleeting | MPQ |
| My pain is splitting | My pain is light and dull | MPQ |
| My pain tiring-exhausting | My pain is bearable | MPQ |
| My pain is sickening | My pain is tolerable | MPQ |
| My pain is fearful | My pain is understandable | MPQ |
| My pain is punishing-cruel | My pain is manageable | MPQ |
| I have a high-level pain | I have a low-level pain | Pain Variability |
| I am in pain | I have no pain | Pain Variability |
| My pain is all that I can think about | My pain is the last thing on my mind | PCS |
| My pain is so severe that is hard to think of anything else | My pain is so mild that I can easily focus on other things. | PCS |
| I am in pain all of the time. | I am pain-free most of the time | PCS |
| I think about my pain all of the time | I rarely think about my pain | PCS |
| I think about my pain most of the time | I seldom think about my pain. | PCS |
| I am constantly aware of my pain | My pain is hardly noticeable | PCS |
| My life lacks purpose | My life is meaningful | PROMIS Meaning and Purpose |
| I am unable to focus | I am able to focus | PROMIS-Pain Interference |
| I am in bed and cannot move because of my pain | I am in bed finding comfort despite my pain | RMDQ |
| I rate my quality of life as poor | I rate my quality of life as good | WHOQOL |
| I am dissatisfied with my health | I am satisfied with my health | WHOQOL |
| I feel unsafe in my life | I feel safe in my life | WHOQOL |
| My physical environment is unhealthy | My physical environment is healthy | WHOQOL |
| I am dissatisfied with my ability to perform my daily living activities | I am satisfied with my ability to perform my daily living activities | WHOQOL |
| I am dissatisfied with my capacity for work | I am satisfied with my capacity for work | WHOQOL |
| I am dissatisfied with myself | I am satisfied with myself | WHOQOL |
| I am dissatisfied with my personal relationships | I am satisfied with my personal relationships | WHOQOL |
| I am dissatisfied | I am satisfied | WHOQOL |
| *Abbreviations:* WHOQOL, World Health Organization quality of life | | |

**Table S4**. Relationship between education years, interview features and semantic embeddings of anchor sentences.

| **Measure** | *ρ* | **p-value** |
| --- | --- | --- |
| Character count | 0.29 | 0.214 |
| Word Count | 0.26 | 0.263 |
| Interviewer duration (min) | **-0.48** | **0.034** |
| Participant duration (min) | 0.2 | 0.410 |
| Total time (min) | 0.12 | 0.616 |
| NRS Pain | -0.22 | 0.360 |
| VAS Pain | -0.29 | 0.219 |
| My pain is throbbing | -0.23 | 0.336 |
| My pain is shooting | 0.06 | 0.802 |
| My pain is stabbing | -0.18 | 0.460 |
| My pain is sharp | -0.21 | 0.381 |
| My pain is cramping | -0.06 | 0.802 |
| My pain is gnawing | 0.27 | 0.241 |
| My pain is hot-burning | -0.04 | 0.854 |
| My pain is aching | 0.29 | 0.214 |
| My pain is splitting | -0.16 | 0.502 |
| My pain tiring-exhausting | 0.22 | 0.363 |
| My pain is sickening | 0.16 | 0.513 |
| My pain is fearful | 0.01 | 0.960 |
| My pain is punishing-cruel | -0.19 | 0.419 |
| I have a high level pain | 0.28 | 0.234 |
| I am in pain | 0.24 | 0.302 |
| My pain is all that I can think about | -0.13 | 0.581 |
| My pain is so severe that is hard to think of anything else | 0.19 | 0.429 |
| I am in pain all of the time. | 0.23 | 0.336 |
| I think about my pain all of the time | 0.03 | 0.894 |
| I think about my pain most of the time | 0.05 | 0.828 |
| I am constantly aware of my pain | 0.08 | 0.726 |
| My life lacks purpose | 0.02 | 0.947 |
| I am unable to focus | 0.23 | 0.336 |
| I am in bed and cannot move because of my pain | -0.38 | 0.100 |
| I rate my quality of life as poor | **0.49** | **0.027** |
| I am dissatisfied with my health | 0.39 | 0.093 |
| I feel unsafe in my life | 0.09 | 0.713 |
| My physical environment is unhealthy | **0.60** | **0.005** |
| I am dissatisfied with my ability to perform my daily living activities | 0.37 | 0.108 |
| I am dissatisfied with my capacity for work | 0.33 | 0.149 |
| I am dissatisfied with myself | 0.13 | 0.581 |
| I am dissatisfied with my personal relationships | 0.37 | 0.104 |
| I am dissatisfied | -0.18 | 0.450 |

**Table S5.** Example semantic similarity scores between anchor sentences and interview sentences. The “interview sentences” are taken from actual interviews performed in this study

### **Anchor Sentence Interview Sentence Semantic Similarity**

I had a huge impact on my life. 0.68

How I live things. 0.56

My life is meaningful

Depending on what level it is, it definitely affects it.

My 11-year-old doesn’t know what cold water is and she was swimming all the way.

Sometimes past, when people are doing stuff, you’re not participating.

0.32

-0.05

-0.05

I haven’t missed a lot of work because of it. 0.65

I am satisfied with my capacity for work

I’m doing well because my functioning as an I’m still a parent.

I still do all the things that I need to get done in my life.

0.65

0.61

Oh wow. 0.36

I work in downtown Rochester, I own a busi- ness, I mess in startup companies and I work out in office largely but I’m fairly active.

0.36

I’ve tried to tell her, I don’t know about it. -0.02

O my habits change from Mario Brothers one day to Taylor Swift the next, to living with her child.

-0.0

**Table S6.** Statistical results of the Kolmogorov – Smirnov tests for the anchors and their antitheses sentences

| **Anchor** | **Antithesis** | **Source** | **KS** | **p-value** | **q-value** |
| --- | --- | --- | --- | --- | --- |
| My pain is cramping | My pain is relaxing | MPQ | **0.95** | **5.80E-10** | **1.92E-08** |
| My pain is splitting | My pain is light and dull | MPQ | **0.80** | **1.33E-06** | **1.46E-05** |
| My pain is throbbing | My pain is steady | MPQ | **0.65** | **2.70E-04** | **2.23E-03** |
| My pain is stabbing | My pain is dull and aching | MPQ | **0.65** | **2.70E-04** | **2.23E-03** |
| My pain tiring-exhausting | My pain is bearable | MPQ | **0.55** | **3.97E-03** | **2.18E-02** |
| My pain is shooting | My pain is dull and localized | MPQ | 0.50 | 1.23E-02 | 5.07E-02 |
| My pain is aching | My pain is fleeting | MPQ | 0.50 | 1.23E-02 | 5.07E-02 |
| My pain is sickening | My pain is tolerable | MPQ | 0.45 | 3.35E-02 | 7.91E-02 |
| My pain is punishing-cruel | My pain is manageable | MPQ | 0.45 | 3.35E-02 | 7.91E-02 |
| My pain is fearful | My pain is understandable | MPQ | 0.40 | 8.11E-02 | 1.67E-01 |
| My pain is sharp | My pain is dull | MPQ | 0.25 | 5.71E-01 | 8.57E-01 |
| My pain is gnawing | My pain is mild and intermittent | MPQ | 0.25 | 5.71E-01 | 8.57E-01 |
| My pain is hot-burning | My pain is numbing | MPQ | 0.20 | 8.32E-01 | 1.00E+00 |
| I am in pain | I have no pain | Pain Variability | **0.85** | **1.43E-07** | **2.37E-06** |
| I have a high-level pain | I have a low level pain | Pain Variability | 0.15 | 9.83E-01 | 1.00E+00 |
| My pain is all that I can think about | My pain is the last thing on my mind | PCS | 0.50 | 1.23E-02 | 5.07E-02 |
| My pain is so severe that is hard to think of anything else | My pain is so mild that I can easily focus on other things. | PCS | 0.50 | 1.23E-02 | 5.07E-02 |
| I think about my pain most of the time | I seldom think about my pain. | PCS | 0.40 | 8.11E-02 | 1.67E-01 |
| I think about my pain all of the time | I rarely think about my pain | PCS | 0.30 | 3.36E-01 | 6.15E-01 |
| I am constantly aware of my pain | My pain is hardly noticeable | PCS | 0.20 | 8.32E-01 | 1.00E+00 |
| I am in pain all of the time. | I am pain-free most of the time | PCS | 0.15 | 9.83E-01 | 1.00E+00 |
| I am unable to focus | I am able to focus | PROMIS -pain interference | **0.55** | **3.97E-03** | **2.18E-02** |
| My life lacks purpose | My life is meaningful | PROMISE-Meaning and Purpose | 0.15 | 9.83E-01 | 1.00E+00 |
| I am in bed and cannot move because of my pain | I am in bed finding comfort despite my pain | RMDQ | 0.30 | 3.36E-01 | 6.15E-01 |
| I rate my quality of life as poor | I rate my quality of life as good | WHOQOL | 0.50 | 1.23E-02 | 5.07E-02 |
| I am dissatisfied | I am satisfied | WHOQOL | 0.50 | 1.23E-02 | 5.07E-02 |
| I feel unsafe in my life | I feel safe in my life | WHOQOL | 0.30 | 3.36E-01 | 6.15E-01 |
| I am dissatisfied with myself | I am satisfied with myself | WHOQOL | 0.30 | 3.36E-01 | 6.15E-01 |
| I am dissatisfied with my personal relationships | I am satisfied with my personal relationships | WHOQOL | 0.25 | 5.71E-01 | 8.57E-01 |
| I am dissatisfied with my health | I am satisfied with my health | WHOQOL | 0.20 | 8.32E-01 | 1.00E+00 |
| My physical environment is unhealthy | My physical environment is healthy | WHOQOL | 0.20 | 8.32E-01 | 1.00E+00 |
| I am dissatisfied with my ability to perform my daily living activities | I am satisfied with my ability to perform my daily living activities | WHOQOL | 0.20 | 8.32E-01 | 1.00E+00 |
| I am dissatisfied with my capacity for work | I am satisfied with my capacity for work | WHOQOL | 0.15 | 9.83E-01 | 1.00E+00 |

**Table S7.** List of correlations between anchor embeddings and NRS and VAS.

|  |  | **NRS** | | | **VAS** | | |
| --- | --- | --- | --- | --- | --- | --- | --- |
| **Anchor** | **Source** | *ρ* | **p-value** | **q-value*** | *ρ* | **p-value** | **q-value*** |
| My pain is steady | MPQ | -0.291 | 0.213 | 0.523 | -0.213 | 0.367 | 0.691 |
| My pain is dull and localized | MPQ | -0.101 | 0.671 | 0.835 | -0.161 | 0.499 | 0.732 |
| My pain is dull and aching | MPQ | -0.238 | 0.313 | 0.666 | -0.172 | 0.469 | 0.732 |
| My pain is dull | MPQ | -0.046 | 0.846 | 0.900 | 0.028 | 0.907 | 0.943 |
| My pain is relaxing | MPQ | -0.094 | 0.694 | 0.848 | -0.073 | 0.759 | 0.864 |
| My pain is mild and intermittent | MPQ | -0.008 | 0.972 | 0.972 | 0.026 | 0.915 | 0.943 |
| My pain is numbing | MPQ | -0.216 | 0.359 | 0.691 | -0.253 | 0.281 | 0.619 |
| My pain is fleeting | MPQ | 0.229 | 0.331 | 0.682 | 0.151 | 0.526 | 0.739 |
| My pain is the last thing on my mind | MPQ | -0.194 | 0.413 | 0.694 | -0.196 | 0.408 | 0.694 |
| My pain is light and dull | MPQ | 0.056 | 0.813 | 0.890 | 0.054 | 0.823 | 0.89 |
| My pain is bearable | MPQ | -0.287 | 0.221 | 0.523 | -0.369 | 0.110 | 0.360 |
| My pain is tolerable | MPQ | -0.300 | 0.199 | 0.523 | -0.369 | 0.109 | 0.360 |
| My pain is understandable | MPQ | -0.220 | 0.352 | 0.691 | -0.161 | 0.497 | 0.732 |
| My pain is manageable | MPQ | -0.118 | 0.62 | 0.827 | -0.196 | 0.408 | 0.694 |
| I have no pain | Pain Variability | -0.156 | 0.513 | 0.736 | -0.412 | 0.071 | 0.272 |
| I have a low level pain | Pain Variability | -0.104 | 0.661 | 0.835 | -0.166 | 0.485 | 0.732 |
| My pain is so mild that I can easily focus on other things. | PCS | -0.085 | 0.720 | 0.864 | -0.056 | 0.815 | 0.89 |
| I am pain-free most of the time | PCS | -0.325 | 0.161 | 0.463 | -0.341 | 0.142 | 0.425 |
| I rarely think about my pain | PCS | -0.018 | 0.942 | 0.956 | -0.075 | 0.755 | 0.864 |
| I seldom think about my pain. | PCS | -0.107 | 0.654 | 0.835 | -0.194 | 0.411 | 0.694 |
| My pain is hardly noticeable | PCS | -0.143 | 0.547 | 0.752 | -0.173 | 0.465 | 0.732 |
| My life is meaningful | PROMIS-Meaning and Purpose | -0.281 | 0.23 | 0.523 | -0.191 | 0.421 | 0.694 |
| I am able to focus | PROMIS-Pain Interference | -0.408 | 0.074 | 0.272 | -0.32 | 0.169 | 0.464 |
| I am in bed finding comfort despite my pain | RMDQ | -0.116 | 0.627 | 0.827 | -0.074 | 0.757 | 0.864 |
| I rate my quality of life as good | WHOQOL | -0.511 | 0.021 | 0.127 | -0.482 | 0.031 | 0.147 |
| I am satisfied with my personal relationships | WHOQOL | -0.545 | 0.013 | 0.085 | -0.503 | 0.024 | 0.130 |
| I am satisfied | WHOQOL | -0.447 | 0.048 | 0.198 | -0.281 | 0.23 | 0.523 |
| I am satisfied with my health | WHOQOL | **-0.614** | **0.004** | **0.043** | **-0.621** | **0.003** | **0.043** |
| I feel safe in my life | WHOQOL | -0.450 | 0.047 | 0.198 | -0.364 | 0.115 | 0.36 |
| My physical environment is healthy | WHOQOL | -0.578 | 0.008 | 0.072 | -0.552 | 0.012 | 0.085 |
| I am satisfied with my ability to perform my daily living activities | WHOQOL | **-0.633** | **0.003** | **0.043** | -0.554 | 0.011 | 0.085 |
| I am satisfied with my capacity for work | WHOQOL | **-0.694** | **< 0.001** | **0.043** | **-0.641** | **0.002** | **0.043** |
| I am satisfied with myself | WHOQOL | **-0.622** | **0.003** | **0.043** | -0.495 | 0.026 | 0.134 |
| *, FDR correction; significant correlations are bolded (q < 0.05). | | | | | | | |
